# A comparison of titers of anti-*Brucella* antibodies of naturally infected and healthy vaccinated cattle by standard tube agglutination test, microtiter plate agglutination test, indirect hemagglutination assay, and indirect enzyme-linked immunosorbent assay

**DOI:** 10.14202/vetworld.2016.717-722

**Published:** 2016-07-12

**Authors:** Anju Mohan, Hari Mohan Saxena, Puneet Malhotra

**Affiliations:** 1 Department of Veterinary Microbiology, College of Veterinary Science, Guru Angad Dev Veterinary and Animal Sciences University, Ludhiana - 141 004, Punjab, India; 2 Department of Animal Genetics and Breeding, College of Veterinary Science, Guru Angad Dev Veterinary and Animal Sciences University, Ludhiana - 141 004, Punjab, India

**Keywords:** antibody titers, *Brucella*, brucellosis, *Brucella abortus* S19 vaccine, bovine brucellosis

## Abstract

**Aim:**

We determined the antibody response in cattle naturally infected with brucellosis and normal healthy adult cattle vaccinated during calf hood with strain 19.

**Materials and Methods:**

The antibody titers were measured by standard tube agglutination test (STAT), microtiter plate agglutination test (MAT), indirect hemagglutination assay (IHA), and indirect enzyme-linked immunosorbent assay (iELISA) as per standard protocols.

**Results:**

The mean STAT titers were 1.963±0.345 in infected cattle and 1.200±0.155 in healthy vaccinated cattle. The difference was extremely significant (p<0.0001). The mean MAT titers were 2.244±0.727 in infected cattle and 1.200±0.155 in healthy vaccinated cattle. The difference was very significant (p<0.005). The mean IHA titers in infected cattle were 2.284±0.574, and those in healthy vaccinated cattle were 1.200±0.155. The difference was extremely significant (p=0.0002). However, the difference in mean iELISA titers of infected cattle (1.3678±0.014) and healthy vaccinated cattle (1.367±0.014) was non-significant. The infected animals showed very high titers of agglutinating antibodies compared to the vaccinated animals. However, it cannot be ascertained whether these antibodies are due to vaccine or response to infection. Since the infected animals had been vaccinated earlier, the current infection may suggest that vaccination was unable to induce protective levels of antibody. The heightened antibody response after infection may also indicate a secondary immune response to the antigens common to the vaccine strain and wild *Brucella* organisms.

**Conclusion:**

The brucellosis infected animals showed very high titers of agglutinating antibodies compared to the vaccinated animals.

## Introduction

Brucellosis is a major bacterial zoonosis of global importance. Brucellosis occurs worldwide but is much controlled in developed countries by routine screening of domestic animals and vaccination program. Clinical disease is still common in Middle East, Asia, Africa, South and Central America, the Mediterranean Basin, and the Caribbean. About 500,000 cases of human brucellosis are estimated to occur worldwide every year. It causes heavy economic loss to the animal industry through abortion, delayed conception, and temporary or permanent infertility in the affected animals [1].

Bovine brucellosis is endemic in all states of India. In India, the occurrence of brucellosis is to the extent of 10% in the marginal herds and 50% in organized farms, and the socio-economic impact of the disease was estimated to run over Rs. 500 crores annually. In Punjab, overall 17.7% prevalence of brucellosis was reported in cattle and buffaloes [2,3]. Brucellosis in animals is clinically characterized by late-term abortions and retention of placenta in females and orchitis and epididymitis in males, with excretion of organisms in semen, uterine discharges, and in milk [4]. Once infected, the animal may continue to shed bacteria and remains a source of infection to others for long period [3]. Mass vaccination is crucial for the control and eradication of bovine brucellosis. The widely used vaccine against brucellosis is derived from the smooth live vaccine strain (S19) for cattle. Although it has some undesirable traits, it has proven to be very useful under most conditions [5-7]. *Brucella abortus* S19 has been effective for the control of brucellosis in adult bovines and preventing abortion as well as decreasing the prevalence in herds [8].

The antibodies induced by vaccination interfere in serological diagnosis of brucellosis. Little data are available in the published literature on comparison of antibody titers due to vaccination and those due to natural infection in cattle. The present study was, therefore, undertaken to explore this aspect of serology of bovine brucellosis.

## Materials and Methods

### Ethical approval

All the experimental protocols performed on cattle were approved by the Institutional Animal Ethics Committee (IAEC). Animals were kept in IAEC approved facilities and received feed and water *ad libitum*.

### Infected and vaccinated cattle

A total of 15 naturally infected brucellosis positive adult cattle, which were vaccinated during calf hood with *B. abortus* strain 19 vaccine (Bruvax; Indian Immunologicals), and 6 normal healthy calf hood vaccinated Holstein-Friesian crossbred adult cattle maintained at the University Dairy Farm were included in the study.

### B. abortus strain 19 (vaccine strain)

The standard vaccine strain *B. abortus* strain 19, procured from the Biological Standardization Division, IVRI, Izatnagar, was used in the present study.

### Collection of serum

Blood samples were collected from cattle through jugular vein for obtaining sera for studying the humoral immune response of the animals. Sera were separated and stored at −20°C until further use.

### Analysis of immune responses

#### Rose Bengal plate agglutination test (RBPT)

Equal volumes (10 µl each) of RBPT colored antigen (Punjab Veterinary Vaccine Institute, Ludhiana) and test serum were mixed on a clean glass slide [9] with the help of a sterilized toothpick. The slide was observed for 4 min for the formation of clumps. The formation of clumps was considered a positive test, whereas the absence of clear clumps was considered a negative reaction.

#### Estimation of antibody titers by standard tube agglutination test (STAT)

The standard OIE method [10] was followed (Table-1). The highest serum dilution showing 50% agglutination was taken as the end point for the titer. A titer of 1:40 or above was considered positive.

**Table-1:** STAT protocol.

Tube No.	Carbol saline (in ml)	Test serum (in ml)	*B. abortus* plain antigen (in ml)	Final dilution
1	0.8	0.2	0.5	1:10
2	0.5	Serial dilution was performed after thorough mixing. 0.5 ml of the contents was transferred from tube No. 1 to the next tube up to tube No. 7. Finally, 0.5 ml of the contents was discarded from tube No. 7	0.5	1:20
3	0.5		0.5	1:40
4	0.5		0.5	1:80
5	0.5		0.5	1:160
6	0.5		0.5	1:320
7	0.5		0.5	1:640
8	1.25		0.75	
9	1.50		0.50	
10	1.75		0.25	

STAT=Standard tube agglutination test, *B. abortus*=*Brucella abortus*

#### Controls

1: Tube No. 08 - 25% agglutination
2: Tube No. 09 - 50% agglutination
3: Tube No. 10 - 75% agglutination.

### Microtiter plate agglutination test (MAT)

MAT was performed as per the method reported earlier [11].

#### Procedure

- Serum samples were serially diluted two-fold in a final volume of 100 µl in 96 well U-bottom microtiter plate (Tarsons)
- Equal volume of 100 µl *B. abortus* plain antigen (Punjab Veterinary Vaccine Institute, Ludhiana) was added to each well. Negative control well containing 100 µl of sterilized normal saline solution (NSS) and 100 µl of the antigen was also kept
- The plate was covered with a lid and incubated at 37°C for 24 h followed by incubation at 4°C for 1 h
- The formation of matt signified agglutination while button formation was indicative of a negative reaction. Titers (log_10_ values) were recorded as the reciprocal of the highest dilution of the serum giving at least 50% agglutination.

### Indirect hemagglutination assay (IHA)

The method reported earlier [12] was followed with minor modifications.

#### Fixation of sheep red blood cells (sRBCs)

Sheep blood was collected aseptically by jugular vein puncture into Alsever’s solution (1:1) and kept at 4°C for 7 days before further processing. The blood was then centrifuged at 1500 rpm for 10 min to pack the erythrocytes. The packed RBCs were washed three times with 5-6 volumes of chilled NSS by centrifugation. Finally, a 10% (v/v) suspension of RBCs was prepared in chilled NSS and stored at 4°C.

#### Fixation and treatment of sRBCs with tannic acid

- A 1% v/v solution of glutaraldehyde was prepared in NSS and stored at 4°C. Equal volumes of chilled glutaraldehyde solution and 10% washed sRBC suspension were mixed and allowed to stand at 4°C for 30 min with intermittent gentle stirring.
- The sensitized sRBCs were packed by centrifugation at 1500 rpm for 10 min at room temperature followed by three washes in NSS to remove free glutaraldehyde and resuspended in the same buffer containing 0.1% sodium azide to yield a 10% suspension of sRBCs. The glutaraldehyde fixed sRBCs (G-sRBCs) were then stored at 4°C.
- A 10% suspension of G-sRBCs was mixed with an equal volume of phosphate-buffered saline (PBS) containing 0.005% tannic acid (w/v) and incubated at 37°C with occasional shaking. The tanned G-sRBCs (TG-sRBCs) were pelleted by centrifugation at 650 ×*g* for 10 min at room temperature and washed three times with PBS to yield a 10% suspension.

### Preparation of antigen

The antigen prepared as described earlier was heated at 56°C for 30 min in a water bath with frequent shaking. Heat treated suspension was then centrifuged at 8000 rpm for 15 min at 4°C. The clear supernatant was separated and stored at −20°C until use.

#### Sensitization of TG-sRBCs with antigen

- One volume of packed RBCs and 15 volumes of the antigen were mixed and incubated for 1-2 h at 37°C in a water bath with frequent shaking
- The sensitized cells thus prepared were washed 3 times with NSS by centrifugation at 2000 rpm for 5 min. After the final wash, packed cells were resuspended in chilled NSS to obtain 1% suspension.

#### Adsorption of serum samples

- To remove the heterophile antibodies, all the test serum samples (3 volumes) were adsorbed with packed sRBCs (1 volume) for 2 h at 37°C with periodic shaking before the test proper. The RBCs were removed by centrifugation
- The suspension was centrifuged at 600 ×*g* for 15 min at 4°C in a refrigerated centrifuge. The supernatant was collected and used for the test.

#### Test protocol for IHA

- PBS (160 µl) and inactivated adsorbed serum (40 µl) were added to the first well (1 in 4 dilution), and 100 µl of PBS was added to all the other wells of a 96 well U-bottom microtiter plate (Tarsons). Two-fold serial dilutions of serum were made in a final volume of 100 µl
- An equal volume (100 µl) of the 0.5% sensitized RBC suspension was added to all the wells. The plates were shaken and left at room temperature for 2 h. Coarse agglutination of RBCs (matt formation) indicated a positive result and formation of a small button of deposited cells was considered as a negative result.

#### Controls

The following three controls were included with each test:

- Antigen control: 100 µl of sensitized and adsorbed RBCs
- RBC control: 100 µl of 1:4 dilution of serum and 100 µl of sensitized RBCs
- Serum control: 100 µl of untreated erythrocytes and 100 µl of test sera.

### Enzyme-linked immunosorbent assay (ELISA)

The serum samples of cattle were tested using Brucellosis Serum ELISA test kit (Idexx). The kit is based on indirect ELISA (iELISA) using inactivated antigen of *B. abortus*. The binding of the antibodies in cattle serum samples with precoated inactivated antigen on microtiter plate is detected by peroxidase-labeled anti-ruminant immunoglobulin G (IgG). The degree of the color that develops (optical density [OD] measured at 450 nm) is directly proportional to the amount of antibody specific for *B. abortus* present in the sample. The diagnostic relevance of the result is obtained by comparing the OD in wells containing the samples with the OD from wells containing the positive control. Antibody titers were calculated using an equation of regression.

#### Procedure

All reagents were thawed to 25°C and mixed by gentle vortexing before use.

- Dispensed 90 µl of diluted wash solution (1:10) into each well of the microtiter plate
- Added 10 µl of the undiluted serum samples and controls into the appropriate wells of the microtiter plate making the final dilution 1:10
- Mixed the contents within each well by gently shaking the microtiter plate
- Covered the microtiter plate with a lid and incubated for 60 min at 37°C in a humid chamber
- Washed each well with approximately 300 µl wash solution 3 times. Aspirated liquid contents of all the wells after each wash. Following the final aspiration, firmly tapped the residual wash fluid from each plate onto absorbent material. Drying of plate between washes and before the addition of the next reagent was avoided
- Dispensed 100 µl conjugate into each well
- Covered and incubated the microtiter plate for 60 min at 37°C in a humid chamber
- Washed each well and aspirated the liquid contents of all the wells after each wash
- Dispensed 100 µl of 3,3’,5,5’-Tetramethylbenzidine substrate into each well and incubated the substrate at 18-26°C for 15 min
- Stopped the color reaction by adding 100 µl of stop solution per well
- The OD was recorded in an ELISA reader at a wavelength of 450 nm.

### Calculation of antibody titers

Antibody titer (Log_10_) Y= a + bx

Where, constant a=1.35; constant b=0.05; X=OD value of a test well/Mean +3 standard deviation value of negative control wells. The standard error of the Y estimate (antibody titer) was + 0.19 log_10_.

### Statistical analysis of data

Data pertaining to antibody titers by STAT, MAT, IHA, and iELISA were statistically analyzed by analysis of variance and t-test.

## Results and Discussion

**Figure-1:**
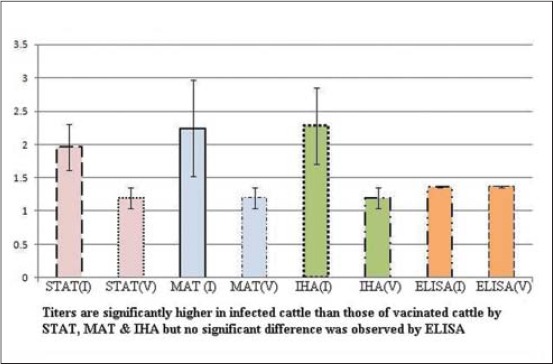
Antibody titers in brucellosis infected or vaccinated cattle by standard tube agglutination test, microtiter plate agglutination test, indirect hemagglutination assay test, and indirect enzyme-linked immunosorbent assay.

Antibody titers of infected or vaccinated cattle were estimated by STAT, MAT, IHA, and iELISA (Tables-2 and 3, Figure-1). STAT revealed the mean titers in infected cattle to be 1.963±0.345, and the corresponding values in healthy vaccinated cattle were 1.200±0.155. The difference was extremely significant (p<0.0001). The mean MAT titers in infected cattle were 2.244±0.727, and the corresponding values in healthy vaccinated cattle were 1.200±0.155. The difference was very significant (p<0.005). IHA revealed the mean titers in infected cattle to be 2.284±0.574, and the corresponding values in healthy vaccinated cattle were 1.200±0.155. The difference was extremely significant (p=0.0002). Interestingly, the difference between the mean iELISA titers of infected cattle (1.3678±0.014) and healthy vaccinated cattle (1.367±0.014) was non-significant.

**Table-2:** Antibody titers of cattle naturally infected with brucellosis.

S. No.	Antibody titers (log_10_)			

	STAT	MAT	IHA	iELISA
1	1.903	2.204	2.204	1.4259
2	2.204	3.107	3.408	1.4328
3	1.602	3.408	2.505	1.4296
4	1.903	3.107	3.107	1.4264
5	1.602	1.602	1.602	1.3615
6	2.204	1.602	1.903	1.4226
7	2.806	3.709	3.107	1.4243
8	1.903	2.204	2.505	1.4251
9	1.602	1.903	1.602	1.3878
10	2.204	1.602	2.505	1.4539
11	1.602	1.903	1.903	0.6101
12	1.602	1.602	1.602	1.2935
13	2.204	1.602	1.903	0.4963
14	1.903	1.903	2.204	1.4084
15	2.204	2.204	2.204	1.4732
Mean±SD	1.963±0.345	2.244±0.727	2.284±0.574	1.3678±0.014

SD=Standard deviation, STAT=Standard tube agglutination test, MAT=Microtiter plate agglutination test, IHA=Indirect hemagglutination assay test, iELISA=Indirect enzyme-linked immunosorbent assay

**Table-3:** Antibody titers of healthy cattle vaccinated with *B. abortus* strain 19 vaccine.

S. No.	Antibody titers (log_10_)			

	STAT	MAT	IHA	iELISA
1	1.301	1.301	1.301	1.361
2	1.301	1.301	1.301	1.361
3	1.301	1.301	1.301	1.361
4	1.000	1.000	1.000	1.361
5	1.000	1.000	1.000	1.367
6	1.301	1.301	1.301	1.396
Mean±SD	1.200±0.155	1.200±0.155	1.200±0.155	1.367±0.014

SD=Standard deviation, STAT=Standard tube agglutination test, MAT=Microtiter plate agglutination test, IHA=Indirect hemagglutination assay test, iELISA=Indirect enzyme-linked immunosorbent assay

Even though a number of antigenic components of *Brucella* have been characterized, the antigen that dominates the antibody response is the lipopolysaccharide (LPS). Numerous outer and inner membrane, cytoplasmic, and periplasmic protein antigens have also been characterized. Some are recognized by the immune system during infection and are potentially useful in diagnostic tests. The L7/L12 ribosomal proteins are important in stimulating cell-mediated responses [13].

Immune response of host to *Brucella* infection is mediated through both humoral and cell-mediated immunity [14]. The role of humoral immunity against intracellular bacterial infections is limited and not protective. Antibody-mediated opsonization by Igs (IgM, IgG1, IgG2a, and IgG3) enhances phagocytic uptake of bacteria, limiting the level of initial infection with *Brucella* but has little effect on the intracellular course of *Brucella* infection [15,16].

*B. abortus* strain 19 is used as a live vaccine and is normally given to female calves aged between 3 and 6 months as a single subcutaneous dose of 5-8 × 10^10^ viable organisms. It is believed to induce protective immunity against *B. abortus* in cattle [17]. However, the effectiveness of this vaccine varies with the age of vaccination, dose, route, and prevalence of brucellosis in the herd [18]. *B. abortus* strain 19 vaccine remains a reference vaccine to which any other vaccine is compared. Persistent antibodies could be detected up to 10-11 months post-vaccination [19].

As evident from our present data, infected animals have very high titers of antibodies compared to the vaccinated animals. However, the high titers do not indicate whether these are protective antibodies due to vaccine or acute response to infection in the absence of a differentiation of infected from vaccinated animals (DIVA) assay or whether they are of any relevance to prognosis. Since the infected animals were the ones who had already been vaccinated during calf hood, the infection in these animals may suggest that the vaccine was unable to induce protective levels of antibody. Second, the heightened antibody response after infection may indicate a secondary immune response to the genus-specific antigens of *Brucella*.

The different titers observed in the same animals by agglutination assays (STAT, MAT, and IHA) and iELISA can be reconciled with the fact that these assays target antigens of different nature, i.e., agglutination assays are directed toward particulate antigens, whereas ELISA detects immune response to soluble antigens. ELISA is generally used to detect IgG antibodies [20]. In brucellosis, specific IgM antibodies dominate during the acute phase of the disease [21]. Specific IgG antibodies are present in the serum of patients at later stages of the illness and in the serum of relapsing patients [22]. ELISA is used to discriminate between the presence of specific IgM and IgG antibodies and to roughly assess the stage of illness [23].

In many countries, STAT is the routine diagnostic test for human and animal brucellosis. It has been reported [24] that STAT has a greater accuracy than that of the RBPT (93.3% and 76.6%, respectively). In a study [25], *Brucella* antibodies were investigated in bovine sera by RBPT, serum agglutination test, MAT, and 2-mercaptoethanol MAT, and MAT was determined as a fast, reliable, and economic test. On evaluation of canine brucellosis by MAT, it was shown [26] that MAT was more sensitive, simpler to perform, and easier than tube agglutination test.

A study [27] has shown that the use of sheep erythrocytes sensitized with a specific LPS antigen in the IHA test provided a specific method, which is more sensitive than the agglutination test. A study [28] was carried out to compare the efficacy of RBPT, STAT, and DotELISA in immunological detection of antibodies to *B. abortus* in sera. The study revealed that DotELISA was the most sensitive of the three tests used. In a study by Ghodasara *et al*. [29], STAT and iELISA were compared for detection of *Brucella* antibodies in cows and buffaloes. The seropositivity was found highest by iELISA (25%) followed by STAT (14.45%). iELISA, RBPT, MAT, and PCR were evaluated [30] for diagnosis of brucellosis in buffaloes, and it was concluded that iELISA detected more samples as positive among these tests. Currently, no DIVA vaccine against brucellosis is available in the market. Identification of extracellular proteins from *Brucella* may aid in discovery of better vaccines or diagnostic molecules [31].

Since cell-mediated immunity is known to play an important role in brucellosis, it would be pertinent to incorporate antigens and adjuvants in the vaccine which could generate cellular immunity of a high protective level to be effective in control of brucellosis.

## Conclusion

We determined the antibody titers by STAT, MAT, IHA, and ELISA in cattle naturally infected with brucellosis and normal healthy adult cattle vaccinated during calf hood with strain 19. The differences between the mean STAT, MAT, and IHA titers of infected cattle and healthy vaccinated cattle were highly significant (p<0.0001, p<0.005, and p=0.0002, respectively). However, the difference in mean iELISA titers of infected cattle and healthy vaccinated cattle was non-significant. The infected animals showed very high titers of agglutinating antibodies compared to the vaccinated animals.

## Authors’ Contributions

AM did the experiments; HMS conceived the idea, planned the study, and wrote manuscript; PM maintained the animals in the farm. Both authors read and approved the final manuscript.
